# Multi-dimensional potential factors influencing COVID-19 vaccine booster acceptance and hesitancy among university academic community in Bangladesh: A cross-sectional comparative study

**DOI:** 10.1371/journal.pone.0281395

**Published:** 2023-04-13

**Authors:** Debendra Nath Roy, Md. Shah Azam, Ekramul Islam

**Affiliations:** 1 Department of Pharmacy, Jashore University of Science and Technology, Jashore, Bangladesh; 2 Department of Marketing, University of Rajshahi, Rajshahi, Bangladesh; 3 Department of Pharmacy, University of Rajshahi, Rajshahi, Bangladesh; Universite Ziane Achour - Djelfa, ALGERIA

## Abstract

**Background and aims:**

Vaccination is the most powerful public health intervention proven to be safe and effective in the battle against the coronavirus disease-2019 (COVID-19) pandemic. Despite the potential therapeutic benefits of primer vaccine dosage regimens, public perceptions of COVID-19 vaccine booster dose (VBD) acceptance and hesitancy vary among various sub-group populations. This study investigates COVID-19 vaccine booster dose acceptance and compares the multi-dimensional potential factors influencing VBD acceptance and hesitancy among university teachers and the student community in Bangladesh.

**Methods:**

This web-based cross-sectional study employed an anonymous, validated, and self-administered questionnaire. The questionnaire items were adopted from a theoretical analysis of the recent relevant literature. The questionnaire was deployed in an on-line-enabled format (Google form) and conveniently distributed to 685 teachers and 990 students between 15th June, 2022 and 15th August, 2022 which resulted in the participation of 1250 (505 teachers vs.745 students) total respondents (response rate 73.72% vs. 75.25%) from various universities in Bangladesh. A non-parametric analytical tool (binary logistic regression) was applied to rationalize the study objectives and a Chi-squared test was performed to estimate the booster- hesitant risky group.

**Results:**

The pooled COVID-19 vaccine booster dose acceptance rates were 84.6% (95% CI 81.5─87.7) and 67.2% (95% CI 63.8─70.6) for teachers and students in the university academic community, respectively. In employing a binary logistic regression, this study revealed that out of twelve (12)multi-dimensional key predictors, “equal safety”, “risk-benefit ratio”, and “variant control” had a significant positive association with VBD acceptance in both sets (p = 0.000, p = 0.000, and p = 0.005, respectively). Varied effects were found for several predictors; post-vaccination “side effects” had a significant negative association (p = 0.020) and “community protection” had significant positive association (p = 0.034) with vaccine booster dose acceptance in the teachers community while these variables were insignificant in the students cohort. “Trust” had a highly significant positive association (p = 0.000);“communication” and “academic attainment” had significant positive associations (p = 0.033 and 0.024, respectively) with VBD acceptance in the students cohort, while these predictors were insignificant in the teachers community. Women were more likely to receive a third dose of the vaccine (OR = 1.4 vs. 0.9 between teacher and student model); however, no significant association between gender and booster vaccine acceptance was found in a comparative Chi-squared model. Therefore, statistically, the booster vaccine-hesitant risky group was not found to implicate the massive booster vaccine drive among the university academic community.

**Conclusions:**

COVID-19 booster vaccine acceptability among the student cohort was slightly lower than pre-roll-out intent. The teacher community was more inclined to get booster vaccinated. Moreover, differences were found between the multi-dimensional potential factors associated with VBD acceptance among teachers and students in university settings. This study explicitly confirmed positive attitudes toward the safety, health benefits, and variants control of the COVID-19 VBD under any circumstances. Post-vaccination side effect concern was found to be a barrier to administering booster shots and a reason for booster skepticism. Tailored communication and health education interventions need to be adopted to improve the public awareness of booster vaccine consequences, and limit booster skepticism.

## Introduction

For three years, the world has battled against the coronavirus disease 2019 (COVID-19) pandemic to protect human lives and adopt new normal lifestyles. The transmission of viral chains can be minimized through adopting host immunity [1] either by massive community vaccination or immunity acquired from previous infections [2]. Several prediction models have been applied and found useful; however, the burden of causalities during the COVID-19 pandemic due to high morbidity and mortality across countries was entirely unpredictable. Hence, the perceived strategy of cost–benefit analysis for achieving acquired immunity via natural infection was not favorable in this instance [3]. Since the disclosure of multiple genomes sequencing of the novel coronavirus as of January 2021, the scientific community has incorporated over 100 potential candidates into the COVID-19 vaccine platform [4]. Worldwide, these vaccines were widely in use by the middle of 2021 and were proven as the best effective therapeutic intervention to prevent coronavirus contaminations, hospitalizations, and unusual deaths [5–7].

Despite all the fortified public health efforts to advance COVID-19 vaccines, recent reports are increasingly documenting a decline in humoral immunity after six months of vaccination with a second dose; it has been increasingly reported that exposure to a new coronavirus variant arrival resulted in the emergence of repeated infection [8,9]. COVID-19 vaccine effectiveness dropped from 74.7% to 53.1% after a few months of nursing home resident vaccination [10]. Due to declining herd immunity and the probable arrival of new coronavirus variants, the World Health Organization (WHO) and scientists around the world decided to promote booster vaccination shots against COVID-19. Recent research reported 11.3-fold and 19.5-fold raised body defense against infection and severe illness among people by administering a COVID-19 booster shot five months after completing the two primer doses [11]. Although booster shots will at some point play a key role in the public health response by preventing infection, hesitancy to receive a booster shot emerged as a vaccination barrier among significant portions of South Asian people [12]. Previous research reported thatCOVID-19 primer vaccine acceptance has declined due to people’s doubtful attitudes toward receiving the vaccine. While the search for coronavirus vaccines was underway, these suspicious outlooks preventing vaccine acceptance were the major barrier to vaccine roll-out and were collectively known as vaccine hesitancy [13,14]. Concerns regarding vaccination hesitancy have intensified as a result of the COVID-19 epidemic. Consequently, it is imperative to decrease COVID-19 vaccine reluctance, which depends on comprehensive insight into its determinants, to mitigate the pandemic. In the current context, the acceptance of primer vaccinations has been shown to differ considerably among various sub-group populations with substantial regional variability [15]. Thus, the rapid roll-out of the third COVID-19 vaccine dose likely faces the same challenges, and low booster dose uptake would be a major hurdle to curbing new corona virus variant arrival globally.

The dynamic relationship between intention and final decision with respect to the COVID-19 booster is beginning to be evaluated in university settings [16–19] and other sub-group populations worldwide [20–26]. As of December 28, 2021, Bangladesh has begun to administer booster doses of the COVID-19 vaccine [27] in the wake of breakthrough infections, the arrival of new variants, and a decline in long-standing protection. Initially, health policy makers decided to boost front-liners and the elderly as a priority group for immunization through VBD. Among different sub-population groups, students are certainly vulnerable to coronavirus exposure due to their active lifestyles, reading in crowded settings, and a perception of disease invulnerability [28]. Moreover, the teacher community plays a significant role in educational attainment, particularly those teachers in boarding facilities. Recent studies have documented the substantial influence of teachers on student attitudes and behavior [29] and dynamic vaccine behaviors existing in a college setting [30]. Several observational studies conducted in Bangladesh to date have focused on COVID-19 vaccination events among diverse sub-group populations [31–38], including university students [39–43]. However; public readiness to accept the COVID-19 vaccine booster dose has rarely been studied in Bangladesh. Hence, we conducted a web-based cross-sectional comparative study focused on COVID-19VBDacceptance among two major and inter-related population sub-groups in Bangladesh. This study thus aimed to investigate COVID-19 VBD acceptance and compare the potential factors influencing VBD acceptance and hesitancy among university teachers and the student community in Bangladesh.

## Materials and methods

We have deposited step-by-step descriptions of the study protocols on *protocols.io* (DOI: doi.org/10.17504/protocols.io.5jyl8jeq6g2w/v1) to enhance the reproducibility of study results.

### Study design

This cross-sectional comparative study used a self-administered, anonymous, and validated multi-item questionnaire to rationalize the study’s outlined objectives. The questionnaire was deployed online using an online survey tool (Google forms) and conveniently sent to teachers and students in different public and private universities between 15^th^June, 2022 and 15^th^August, 2022 using electronic collection methods (social media platform and emails) and following the STROBE guideline.

At the beginning of the questionnaire, the investigators incorporated a separate paragraph describing the study and terms of consent for participating. Hence, completion of the survey by a participant was considered indirect written consent. Permission to conduct this cross-sectional comparative study has been obtained from the **“**Ethical Review Committee” (ERC), Faculty of Biological Science and Technology, Jashore University of Science and Technology in Bangladesh. The detail research protocol was reviewed and evaluated by the ERC before the study began. Data were collected and analyzed anonymously, while no clinical intervention was applied to the subjects. Hence, the Ethical Review Committee of the university approved the study as exempt. There was no external funding.

### Setting and participants

Teachers and students of government-sponsored (public) and non-government-sponsored (private) university settings in Bangladesh were the participants of this comparative analysis. No financial or in-kind reward was offered to participants who completed the survey. According to the latest census, 51 public and 108 private universities are approved by the University Grants Commission (UGC) in Bangladesh.

### Participant’s inclusion criteria

The eligibility criteria for the participants were as follows: (i) understand and agree to the study objectives and provide anonymous data on COVID-19 booster vaccination, (ii) public and/or private university teachers in Bangladesh, and (iii) students currently studying in a Bangladeshi public or private university. This study did not harm participants, and participants were free to reject participation at any time of the study period.

### Measures and survey instrument development

The theoretical concept of COVID-19 vaccine acceptance and hesitancy was conceptualized using recent systematic reviews conducted on the topic [15,44]. The questionnaire key items were adopted from a theoretical analysis of recent studies focusing onCOVID-19 booster consequences across countries[16–26]. Moreover, in-field expert consultation was conducted in formulating the primary questionnaire items. The questionnaire was constructed in the English language, highlighted multi-dimensional aspects of COVID-19 booster vaccination consequences, and was designed to take approximately 15 minutes to complete. Each item in the preliminary questionnaire was content- and face-validated by a panel of public health experts, which ensured the relevance and clarity of the questionnaire. Pilot testing (n = 10+10) was undertaken among the targeted population before administering the ultimate version of the questionnaire to validate the legitimacy and relevance of the instrument. The outcomes of the piloted research design were not taken into account for the final analysis.

The survey instrument assessed (1) socio-demographic characteristics of the respondents; (2) intention to uptake COVID-19 VBD; and (3) key factors influencing COVID-19 VBD acceptance and hesitancy. A non-parametric data analytical tool (binary logistic regression) was employed to analyze the association mode between predictor variables and the outcome variable with a 95% confidence interval (CI).

### Survey administration

The convenience sampling technique was used for systematic data gathering from online survey tools. This process created a survey with the goal of collecting maximum insights from teacher and student samples for the purpose of developing quantitative variables of the attributes. Commencing with a basic description of the study goal and vision, the questionnaire also emphasized that enrollment was purely optional. Identities, birth dates, and personal information were not collected for the survey. Prior to completing the questionnaire form, all participants provided their informed authorization describing the study terms and objectives to participate willingly. Data integrity was maintained by ensuring anonymity throughout the study period and by requesting accurate responses and choices from participants. The online questionnaire was distributed among teachers and students in various universities, who were all, encouraged to participate; thus, a potential source of non-response bias was minimized.

### Study variables

For the response variable of the study, we measured COVID-19 VBD acceptance as a binary variable (1 = Yes, 0 = No). Socio-demographic profiles of the respondents were also captured and expressed in suitable scale. In analyzing the data in the binary regression model, we investigated the impact of several socio-psychological and vaccine-related independent variables on the outcome response variable (VBD acceptance) dichotomized into 1 = Yes and 0 = No.

### Study size

The following SurveyMonkey formula was used to calculate the least required sample size with a confidence interval of 95% (z score of 1.96) and a 5% margin of error.


$$\;Sample\;size\;=\;\frac{\frac{z^{2}\times p(1-p)}{e^{2}}}{\frac{1+\left(z^{2}\times p(1-p)\right)}{e^{2}N}}$$

Where N = population size, p = sample probability, e = margin of error (percentage in decimal form), z = z-score.

In general, the least required 500 data are recommended to conduct binominal regression analysis in observation studies with large sample size that characterize the parameters. Another formula is n = 100+50i where *i* stand for number of independent variables included into the analysis [45–47].We pre-tested data samples (n = 10+10) as a pilot test to examine the instrument’s clarity and to understand the average time spanned need for completing the survey.

### Equations for binominal regression

Binominal regression equations are followings:

$$y=\;\text{Constant}\;(\text{Z})+{\text{b}}_{1}{\text{x}}_{1}+{\text{b}}_{2}{\text{x}}_{2}+{\text{b}}_{3}{\text{x}}_{3}+\ldots \ldots \ldots \ldots \ldots \ldots \ldots .+{\text{b}}_{\text{m}}{\text{x}}_{\text{m}}$$ (1)

where y is the linear combination function.


P=P(Z=1)=Zx/[1+exp(Zx)] (2)

here, *P* represented the probability of vaccine uptake intent and x indicated the vector of independent variable. If function of y is characterized as

$$\text{logit}(P)-\text{y}={\text{log}}_{\text{e}}[P/1-P]=\text{logit}(P)$$ (3)

Usually Eqs (2) and (3) is expressed and written as logit (*P*) or the log odd ratio as follows-

$$\text{logit}(P)={\text{log}}_{\text{e}}[P/1-P]=\text{BX}$$ (4)

where logit(*P*) means log odd ratio.

#### Statistical methods

Descriptive statistics utilized weighted frequencies and percentages of the variables to analyze socio-demographic profiles and categorical variables. A non-parametric data analytical tool called binary logistic regression was employed to explore the pattern of association between explanatory variables and the response variable. All the key assumptions related to binary regression analysis were examined to adjust the model suitability. Assumptions of binary logistic analysis were tested. Raw data were inserted into Microsoft Excel version 10 and imported to Statistical Package for the Social Science (SPSS) software. IBM-SPSS version 25 (RRID: SCR_016479) was used for analyzing the data. In this study analysis, p<0.05 was considered statistically significant. The online survey denied the acceptance of incomplete survey responses; thus, no missing data were analyzed.

## Results

### Respondent’s characteristics

Table 1 displays comparative socio-demographic characteristics among teachers and students. We checked the eligibility criteria and confirmed participant inclusion accordingly through online survey restrictions. A lack of digital devices and limited internet access could be reasons for study non-participation. In total, 505 teachers and 745 students from various universities in Bangladesh were included for final analysis. Most of the teachers were 31–35 years or36-40 years in age range (20.8% and 20.5%, respectively), and 59.5% of students were youth-aged ranging from 21 to 25 years. Although the majorities (63.6%) of teachers were public university faculty, a significant portion 44.7% of students studied in private universities. However, Dhaka division had the highest count of both teachers and students (28.4% vs. 28.5%). Most teachers had completed two doses of vaccines, while this rate was slightly lower for students (91.5% vs. 61%). Appropriately, 29.6% female teachers and 48% female students participated from various universities. Most participants were Muslim by religion (79.4% vs.72.6%), while histories of COVID-19 positive results were (16.2% vs. 22.5%).

**Table 1:** Overview of socio-demographic profile of study participants (N = 505 vs.745).

Variables	Teachers		Students	
	*N*	*%*	*N*	%
**Age distribution**				
16–20Years	0	0	212	28.5
21–25Years	0	0	443	59.5
26–30Years	52	10.3	82	11.0
31–35 Years	105	20.8	8	1.0
36–40 Years	103	20.5	0	0
41–45 Years	91	18.0	0	0
46–50 Years	79	15.6	0	0
51–55 Years	52	10.3	0	0
56–60 Years	17	3.4	0	0
61–65 Years	6	1.1	0	0
**University type**				
Publicly funded	321	63.6	412	55.3
Privately funded	184	36.4	333	44.7
Others	0	0	0	0
**Geographical distribution**				
Dhaka	151	28.4	212	28.5
Rajshahi	70	26.7	95	12.7
Khulna	94	21.3	121	16.3
Chattogram	49	5.7	82	11.0
Mymensingh	34	5.0	76	10.2
Rangpur	32	9.5	57	7.7
Sylhet	41	2.0	61	8.1
Barishal	34	1.3	41	5.5
**Gender**				
Male	356	70.5	387	52.0
Female	149	29.5	358	48.0
**Religion**				
Muslim	401	79.4	541	72.6
Hindu	99	19.6	182	24.4
Others	5	1.0	22	3.0
**Vaccination status**				
Completed two doses	462	91.5	455	61.1
Received 1^st^ dose	36	7.1	209	28.1
Not vaccinated yet	7	1.4	81	10.8
**History of COVID-19**				
Corona infected previously	82	16.2	188	22.5
**Booster dose acceptance**				
Booster shot hesitancy	78	15.4	244	32.8
Intent to receive booster	427	84.6	501	67.2

### Results of descriptive statistics

Table 2 describes the descriptive results of the predictor variables and outcome variable in the study. The outcome event in this analysis was reported as‘COVID-19 vaccine booster dose acceptance’. The pooled COVID-19 VBD acceptance rate was 84.6%, 95% CI 81.5─87.7 in the teacher community and 67.2%, 95% CI 63.8─70.6 in the student cohort. The online survey barred acceptance of any incomplete survey instruments, so missing data were not produced by each variable of interest.

**Table 2:** Descriptive results of the variables.

Variables	Teachers		Students	
	Mean	SD	Mean	SD
I Intend to accept COVID-19 booster dose anytime (Yes = 1, otherwise = 0)	0.86	0.326	0.86	0.328
Booster doses are similarly safe as were previous doses of COVID-19 vaccines (Yes = 1, otherwise = 0)	0.78	0.404	0.82	0.398
Booster doses will produce severe side effects (Yes = 1, otherwise = 0)	0.56	0.498	0.59	0.498
Booster shot has long effectiveness to protect COVID-19 (Yes = 1, otherwise = 0)	0.92	0.314	0.86	0.328
Effective communication on booster vaccine dose is necessary (Yes = 1, otherwise = 0)	0.78	0.432	0.81	0.420
Booster doses provide repeated protection against COVID-19 (Yes = 1, otherwise = 0)	0.52	0.496	0.64	0.488
The therapeutic benefit of booster doses overweigh the risk (Yes = 1, otherwise = 0)	0.59	0.486	0.66	0.471
I receive the vaccine’s booster dose on priority bases to protect the community people from COVID-19 infection (Yes = 1, otherwise = 0)	0.45	0.421	0.46	0.425
I trust that booster doses provides long-term herd immunity (Yes = 1, otherwise = 0)	0.90	0.303	0.87	0.338
Booster doses are required to control the arrival new coronavirus variants (Yes = 1, otherwise = 0)	0.68	0.474	0.64	0.487
I will receive booster to participate face-to face class at my university (Yes = 1, otherwise = 0)	0.90	0.492	0.60	0.396
I have adequate information about COVID-19 booster vaccine doses (Yes = 1, otherwise = 0)	0.79	0.421	0.79	0.406
Vaccination programs at regional, national, and global level encourage me to receive booster doses (Yes = 1, otherwise = 0)	0.74	0.414	0.78	0.438

### Model summary

Table 3 provides a model summary for both participant groups. The joint impact of all the predictor variables on the dependent variable was determined by using a Nagelkerke R squared test that explained the model summary.

**Table 3:** Comparative model summary.

Model Summary					
-2 Log likelihood		Cox & Snell R square		Nagelkerke R square	
Teachers	Students	Teachers	Students	Teachers	Students
240.619^a^	296.087^a^	0.389	0.407	0.584	0.613

In Table 3, the result of a Cox–Snell R squared test indicates that the outcome variables (VBD acceptance) are given as (38.9%─58.4%) vs. (40.7%─61.3%) by the explanatory variables used in the teacher and student models, respectively, which are assumed to be good levels.

### Goodness-of-model fit

The goodness-of-model fit of the model is explained by omnibus tests of model co-efficient and a Hosmer-Lemeshow test in Table 4. We evaluated the assumptions, and the result showed that the significance level (*p*-value) for the omnibus tests of model coefficients is significant (*p<0*.*05*), while it was insignificant (*p*>0.05) in Hosmer-Lemeshow tests for both studied models. These results indicate very good fitness of the study samples for the binary logistic regression.

**Table 4:** Omnibus tests of model coefficients and Hosmer-Lemeshow test.

Omnibus tests of model coefficients							
Chi-square					Significance level		
	Teachers		Students		Teachers		Students
Step	102.408		153.233		0.000		0.000
Block	102.408		153.233		0.000		0.000
Model	102.408		153.233		0.000		0.000
Hosmer-Lemeshow test							
Chi-square				Significance level			
Teachers		Students		Teachers		Students	
8.254		10.658		0.784		0.438	

### Binary logistic regression analysis

Table 5 represents the comparative results of regression analysis. According to the regression models, out of twelve multi-dimensional predictors, “equal safety profile”, “risk–benefit ratio”, and “variant control” had a significant positive association with VBD acceptance in both sets (p = 0.000, p = 0.000, and p = 0.005, respectively). Varied effects were found for several predictors; post-vaccination “side effects” had a significant negative association and “community protection” had a significant positive association (p = 0.020 and p = 0.034, respectively) with vaccine booster dose acceptance in the teacher community, while these variables were insignificant in the student cohort. However, “trust” had a highly significant (p = 0.000) positive association and “communication” and “academic attainment” had a significant positive association (p = 0.033 and 0.024, respectively) with VBD acceptance in the student cohort, while these predictors were insignificant in the teacher community.

**Table 5:** Results of binary logistic regression analysis.

Teachers community					
Variable	B	S.E.	Wald	Sig.	Exp(B)
Constant	2.081	0.619	3.338	0.047	0.439
**Equal safety**	1.671**	0.421	15.769	0.000	5.317
**Side effect**	-0.424*	0.520	1.799	0.020	1.711
Effectiveness	1.966	0.456	18.578	0.497	7.142
Communication	0.248	0.434	0.327	0.567	0.780
Repeated immunity	0.393	0.406	0.936	0.051	1.481
**Risk-benefit ratio**	0.265**	0.390	0.462	0.000	1.303
**Community protection**	1.092*	0.484	5.086	0.034	0.336
Trust	0.112	0.508	0.048	0.826	1.118
**Variant control**	0.214*	0.307	0.876	0.039	2.346
Academic attainment	-0.766	0.357	4.598	0.032	2.152
Information	-0.298	0.421	0.501	0.479	0.742
Vaccine justice	0.568	0.421	0.956	0.785	1.121
Students cohort					
Variable	B	S.E.	Wald	Sig.	Exp(B)
Constant	2.819	0.585	10.077	0.024	0.262
**Equal safety**	1.297**	0.364	12.723	0.000	3.659
Side effect	0.328	0.333	0.968	0.080	1.388
Effectiveness	1.108	0.391	8.027	0.065	3.030
**Communication**	0.987*	0.331	8.886	0.033	2.684
Repeated immunity	0.613	0.322	3.615	0.057	1.845
**Risk-benefit ratio**	0.218**	0.321	0.463	0.000	0.804
Community protection	1.509	0.498	9.194	0.982	4.523
**Trust**	1.021**	0.320	2.147	0.000	2.833
**Variant control**	0.140*	0.331	0.180	0.031	1.151
**Academic attainment**	2.045*	0.657	5.561	0.024	1.021
Information	-0.059	0.348	0.028	0.866	0.943
Vaccine justice	0.678	0.576	4.414	0.954	0.768

note:** = significant at <0.01 and ** = significant at <0.05.

### Pearson’s Chi-squared test results

The following Table 6 represents the results of a Pearson’s Chi-squared test and odds ratio for VBD risky group estimation. The odds for accepting COVID-19 VBD were (1.5 vs. 0.9) between the models; however, Chi-squared test results revealed an insignificant (p>0.05) association between booster acceptance and gender. Hence, statistically, no group (female/male) was found to be a booster-hesitant risky group affecting country-wide booster vaccination implementation in near-real-time.

**Table 6:** Pearson’s Chi-squared test results in a comparative model.

Teachers community											
Chi-square tests											
		Value			Asymptotic significance (2-sided)			Exact sig. (2-sided)			Exact sig. (1-sided)
Pearson chi-square		2.406^a^			0.121						
Continuity correction^b^		1.981			0.159						
Likelihood ratio		2.411			0.121						
Fisher’s exact test								0.150			0.080
Linear-by-linear association		2.401			0.121						
N of valid cases		505									
Risk estimate					Value			95% Confidence Interval			
								Lower			Upper
	Odds ratio for gender: (Female / Male)				1.466			0.886			2.770
	For cohort I intend to accept vaccination anytime = No				1.385			0.897			2.457
	For cohort I intend to accept vaccination anytime = Yes				.848			0.886			1.015
	N of valid cases				505						
Students cohort											
Chi-squared tests											
			Value	Asymptotic significance (2-sided)			Exact sig. (2-sided)			Exact sig. (1-sided)	
Pearson chi-square			0.071^a^	0.790							
Continuity correction^b^			0.019	0.889							
Likelihood ratio			0.071	0.790							
Fisher’s exact test							0.801			0.444	
Linear-by-linear association			0.071	0.790							
N of valid cases			745								
Risk estimate				Value		95% Confidence Interval					
						Lower			Upper		
	Odds ratio for gender: (Female / Male)			0.935		0.569			1.537		
	For cohort I intend to accept vaccination anytime = No			0.943		0.610			1.457		
	For cohort I intend to accept vaccination anytime = Yes			1.008		0.948			1.073		
	N of valid cases			745							

**Fig 1:**
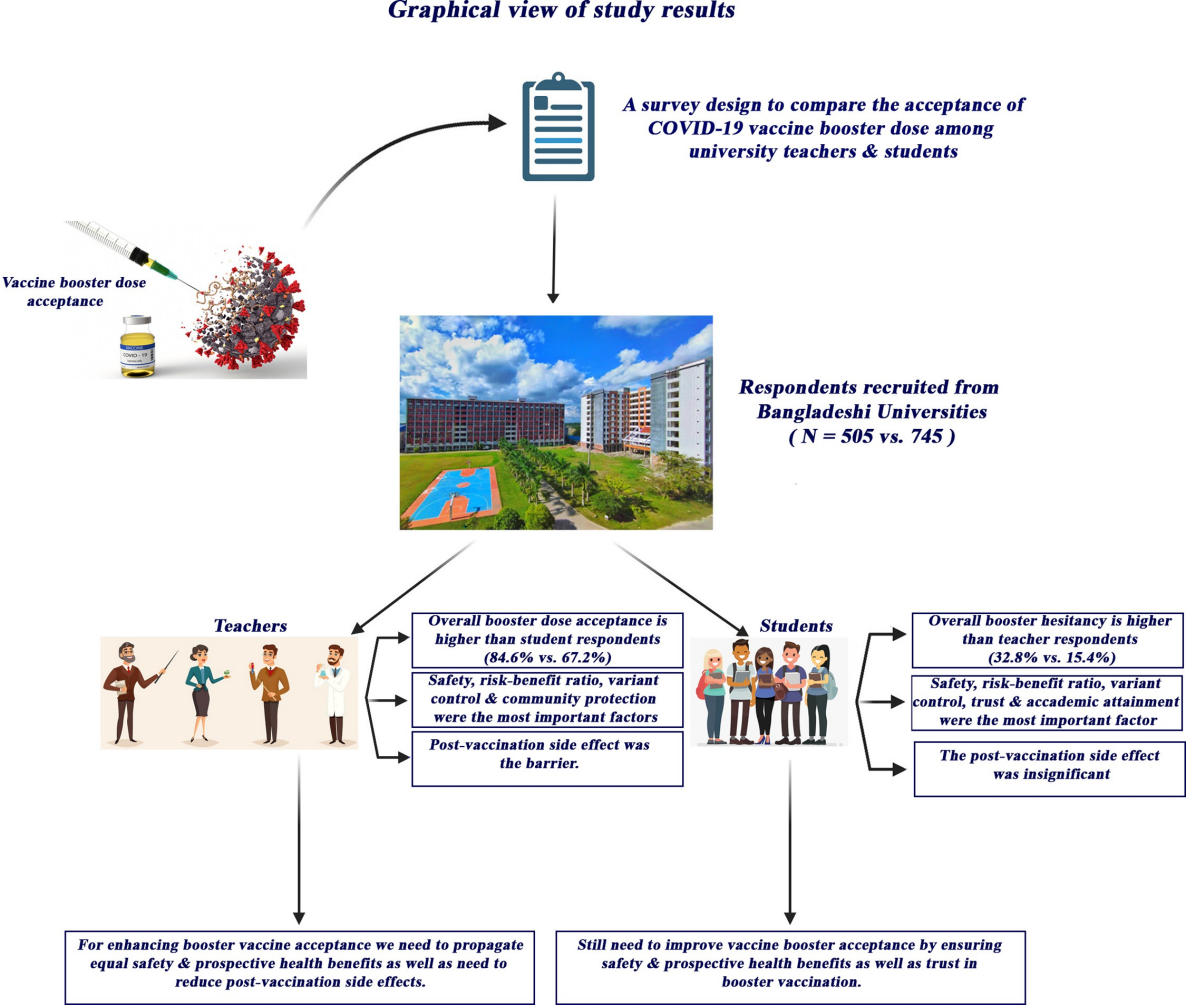
Graphical overview of the study outcomes.

“Fig 1” displays a comparative graphical view of the potential factors influencing COVID-19 vaccine booster acceptance among university academic community in Bangladesh.

## Discussion

One of the most powerful scientific interventions proven to limit several contagious infections and aid in the eradication of diseases is vaccination. The present investigation seeks to assess COVID-19 vaccine booster acceptance and compares multi-dimensional potential factors associated with booster acceptance and hesitancy among teachers and students in university settings. According to our results, the pooled booster vaccine acceptance rate was 84.6% vs. 67.2% for teachers and students, respectively. Appropriately, teachers are more likely to get booster vaccinated against COVID-19 infection. Despite general compliance with the common predictors, we found several differences in the factors associated with VBD acceptance and hesitancy. Equal safety, risk-benefit ratio, and variant control were common factors for VBD acceptance in both groups. Community protection and post-vaccination side effects concern were identified factors among the teacher’s community, while trust, communication, and academic attainment were identified among student cohorts.

Comparable challenges regarding misconceptions and beliefs about the acceptability of the COVID-19 booster vaccine for both adolescents and grownups were observed in the general community. Thus, determining the severity of this challenge and strategizing to lessen booster dose reluctance and get the university community immunized against the coronavirus is imperative. A study conducted on COVID-19 VBD acceptability amongst various sub-group populations reported that in Germany, 87.8% of university students and staff were willing to receive the VBD [16]. Similarly, 71% of adult Poles in Poland [48]; 74.5% of students and health professionals in Poland [17]; 75.3% and 72.7% of the university community in Italy and Belgium, respectively [18,19]; 62% of adult Americans [21]; and 71.3% of healthcare workers in the Czech Republic [49] have declared their willingness to accept the COVID-19 VBD. These results are consistent with our findings. However, low vaccine uptake intention or vaccine apprehension is a complex heterogeneous occurrence that has steadily increased in more than 90% of countries since 2014 [50]. Public acceptance and hesitance towards COVID-19 vaccine has been well-documented for a regularly scheduled dose regimen [15,51,52] as well as for COVID-19 VBD receptivity [22,53,54].

Numerous multi-dimensional psychological concerns have contributed to altered vaccine uptake decisions and determine actual vaccination behavior. The analysis of Roy et al. reported that the most common predictors of vaccine acceptance are safety and efficacy, in other words, trust in the vaccine, but also, at least in Asia, the influence of information, and side effects was significant [15]. Perceived vaccine safety and post-vaccination side effects concern have gradually become the most prominent predictors of primer dose COVID-19 vaccine behavior [51,55–57] as well as booster shot behavior [16,17,49,53].This study also observed that equal safety, risk–benefit ratio, and variant control had a positive association with VBD acceptance in both groups. The outcomes of our research also highlighted post-vaccination side effects as a significant predictor of COVID-19 VBD acceptance among teachers, while this was insignificant in the student set. Although no severe adverse effects were encountered [58,59], moderate side effects were reported during the administration of COVID-19 vaccines for elderly individuals and individuals with a co-morbid diagnosis. University students are youth-aged; adopt active lifestyles, and have faith in disease invulnerability, so the side effects factor is insignificant for this group. Both groups acknowledged that the perceived benefits of the booster dose outweigh the risks and that the booster shot is essential for controlling new variant arrival. However, in this regard, students argued that primer doses are sufficient to provide long-term protection. A recent study deduced that vaccine safety profile, variant control, and risk–benefit ratio were the principal predictive factors regarding VBD acceptance among the university community [17] and general population [22]. In our study, community protection was a significant predictor of VBD acceptance among the teachers, while academic attainment was significant among students. Since teachers are senior citizens, they hold roles of accountability for social and community care over students; hence, community protection has been recognized as an important concern among teachers. Adequate health protection for family members, the community, and patients have been identified as altruistic promoters of VBD acceptance [16]. Moreover, institutional or academic recommendation to uptake the third dose of the vaccine to attain face-to-face classes encourages student booster dose acceptance.

The findings of the present survey demonstrated that trust was an important predictor of booster dose decision among the student cohort. Trust was one of the key determinants of vaccine optimization because confidence regarding vaccine safety, side effects, and efficacy were critically influenced by the level of trust regarding vaccination [60–62]. Tailored and credible health communication has influenced positive health behaviors, guided decision-making, addressed concerns, and built trust in mass vaccination programs [63]. Communication provided by healthcare professionals and government spokes people has been crucial to building public trust for rapid vaccination turn-outs [64]. Higher levels of institutional trust are significantly associated with a greater willingness to uptake two primer vaccine doses [65] and a third booster dose [66]. A growing body of research has noted that receiving two doses provides only limited and short-lived protection against coronavirus infection with the arrival of new variants such as the omicron variant [67]. A study from Saudi Arabia in the first week of omicron spread showed that two-thirds of healthcare workers (HCWs) felt that vaccination was the best option to prevent further spread of the new omicron variant [68]. However, the utility of prospective vaccines to control new coronavirus variants depends on their public acceptance [69]. COVID-19 vaccine acceptance has varied substantially among students in different geographical locations, and numerous multi-dimensional factors are associated with students’ COVID-19 vaccine hesitancy [70]. We have included a large data sample to provide external validity of this study’s results. Substantial variations in respondents’ socio-demographic profiles and a large sample size provided much strength with which to predict the generalizability of the study findings toensureCOVID-19 VBD confidence and receptivity among the general population.

### Implications

This comparative study has some practical implications for policy support, practices, and future research. This study largely benefits health policy makers, health stakeholders, and vaccine promoters in developing evidence-based booster dose promotional planning. Potential factors underlying booster vaccine acceptance and hesitancy would be functional in designing rigorous health interventions involving key messages delivered by community leaders and vaccine policy makers [71]. This study’s findings therefore provide support in overcoming barriers and propagating facilitators while enhancing teacher and student health engagement in a nationwide booster vaccine roll-out; thus, they may help governments to design a booster dose protocol accordingly. In terms of research, this study may act as scientific evidence for initiating further observational studies of COVID-19 vaccine booster acceptance by examining the association between vaccine hesitancy and other confounding variables. Since the pattern of COVID-19 vaccine reluctance can change over time [72], this study assists in the design of a long-term surveillance study for tracking the temporal changes in factors associated with COVID-19 vaccine booster decisions.

## Strengths and limitations

This is the first study focusing onCOVID-19 booster vaccine acceptance, and it applies a new analytical approach to exploring the key determinants of booster vaccine acceptance and hesitancy among two important sub-group populations in university settings. The first strength of our study lies in the fact that its data encompassed people in age groups between 16 and 65 years old; another strength is that a thorough analysis was conducted. Health benefits apparently led participants to uptake VBD over the perceived risk; thus, prospective community benefits outweighing perceived risk have been established in receiving VBD. The significance of community protection over a perception of disease invulnerability was clearly demonstrated to accelerate booster vaccination acceptance. The identification of vaccination trust and academic attainment as a positive predictor for receiving a booster shot adds new value to the existing field of research. The electronic recruitment of participants and the online survey mode provide a rapid strategy with which to obtain a meaningful estimate of effect size and associated variability, which helps researchers to receive a large data sample quickly [73].

The following aspects may have contributed to specific research limitations. Firstly, since this survey was cross-sectional, the causal relationship between the investigated variables could not be validated. The results may be overestimated or underestimated based on self-reported feedback. Secondly, according to the sample size selection criteria, the sample size of our study was inadequate in comparison with the total population size. The electronic data collection mode created the possibility of a non-response bias for those who did not participate in this survey because the digital data collection procedure often fails to capture the depth of information which could have otherwise been possible using an in-person approach. Thirdly, human behavior is transformative and may be altered by changes to the perceived health risk, vaccine characteristics, and vaccine deployment. Fourthly, the sequential alteration of factors associated withCOVID-19 vaccine booster acceptance could still take place, and some additional confounding factors may lead to booster hesitancy that were not defined in this study. These limitations must be addressed in future studies on the topic. Finally, the perceptions to the new omicron and bivalent vaccines were not included in this study.

## Conclusions

The COVID-19immunization actions of the university academic community were evaluated when health policy makers launched a COVID-19vaccine into vaccination programs throughout the country. This study provided insight into the multi-dimensional determinants of booster acceptance among two academically influential groups in universities. Understanding opinions on COVID-19 booster vaccine acceptance and supporting the academic community’s booster vaccine readiness are thus critical for implementing large-scale booster vaccinations. Intention to receive the COVID-19 booster vaccine was slightly lower among students than teachers. This study deduced that several multi-dimensional potential factors were associated with VBD acceptance decisions, and differences were found between teachers and students regarding the potential factors of VBD acceptance. This study confirms the importance of a positive attitude toward the vaccine’s safety profile as were previous doses of COVID-19 vaccines, prospective health benefits, variant control, community protection, trust, health communication, and academic attainment for receiving a booster shot, while post-vaccination side effects concern was a barrier and primary reason for booster dose skepticism. Tailored communication and multi-disciplinary educational intervention must be adopted to improve public adherence and knowledge about booster vaccine consequences and limit booster skepticism.

## Abbreviations

COVID-19: Coronavirus disease-2019
VBD: Vaccine Booster Dose
WHO: World Health Organization
UGC: University Grants Commission
SPSS: Statistical Package of Social Science
LMICs: Low and middle-income countries
